# Evaluation of Community-Based, Mobile HIV-Care, Peer-Delivered Linkage Case Management in Manzini Region, Eswatini

**DOI:** 10.3390/ijerph20010038

**Published:** 2022-12-20

**Authors:** Chutima Suraratdecha, Duncan MacKellar, Thabo Hlophe, Makhosazana Dlamini, Dawud Ujamaa, Sherri Pals, Lenhle Dube, Daniel Williams, Johnita Byrd, Phumzile Mndzebele, Stephanie Behel, Ishani Pathmanathan, Sikhathele Mazibuko, Endale Tilahun, Caroline Ryan

**Affiliations:** 1 Division of Global HIV and TB, Center for Global Health, U.S. Centers for Disease Control and Prevention, Atlanta, GA 30329, USA; 2 Eswatini Ministry of Health, Mbabane P.O. Box 5, Eswatini; 3 Population Services International, Mbabane P.O. Box 170, Eswatini; 4 ICF International, Atlanta, GA 30345, USA; 5 U.S. Centers for Disease Control and Prevention, Pretoria P.O. Box 9536, South Africa; 6 U.S. Centers for Disease Control and Prevention, Mbabane P.O. Box D202, Eswatini

**Keywords:** community-based HIV services, linkage to care, retention to ART, cost, cost-outcome, Eswatini

## Abstract

The success of antiretroviral therapy (ART) requires continuous engagement in care and optimal levels of adherence to achieve sustained HIV viral suppression. We evaluated HIV-care cascade costs and outcomes of a community-based, mobile HIV-care, peer-delivered linkage case-management program (CommLink) implemented in Manzini region, Eswatini. Abstraction teams visited referral facilities during July 2019–April 2020 to locate, match, and abstract the clinical data of CommLink clients diagnosed between March 2016 and March 2018. An ingredients-based costing approach was used to assess economic costs associated with CommLink. The estimated total CommLink costs were $2 million. Personnel costs were the dominant component, followed by travel, commodities and supplies, and training. Costs per client tested positive were $499. Costs per client initiated on ART within 7, 30, and 90 days of diagnosis were $2114, $1634, and $1480, respectively. Costs per client initiated and retained on ART 6, 12, and 18 months after diagnosis were $2343, $2378, and $2462, respectively. CommLink outcomes and costs can help inform community-based HIV testing, linkage, and retention programs in other settings to strengthen effectiveness and improve efficiency.

## 1. Introduction

Antiretroviral therapy (ART) for persons living with HIV (PLHIV) requires continuous engagement in care and adherence to sustain HIV viral load suppression and reduce AIDS-associated mortality and the transmission of HIV. In 2014, the 95-95-95 Fast-track initiative (diagnose 95% of PLHIV; treat 95% of diagnosed PLHIV; and suppress the HIV viral load in 95% of treated PLHIV) was adopted to end the global HIV/AIDS pandemic as a public health threat by 2030 [1]. Eswatini, a country in sub-Saharan Africa with an estimated HIV prevalence of 27% among adults aged 18–49 years, has made substantial progress in scaling up HIV testing services and ART, and in 2019, it was the first country to achieve 95-95-95 [2]. Despite this notable achievement, estimated annual HIV incidence among adults aged 15–49 years in Eswatini remains high (1.2%), underscoring the need for cost-effective HIV prevention and treatment programs to reach and sustain epidemic control [3]. 

To help achieve 95-95-95, the World Health Organization (WHO) recommends implementing several effective community-based HIV testing and ART linkage services [4,5,6]. PLHIV diagnosed in community settings who often are not seeking care when tested are particularly vulnerable to barriers to care, and relatively few initiate ART in health-care facilities soon after diagnosis when provided with a referral as the only linkage service [7,8,9,10]. Two studies in Eswatini conducted during 2011–2013 suggested that only 26–37% of PLHIV are linked to HIV care within 6 months after diagnosis in community settings [9,10]. To improve early linkage to care among community-diagnosed clients in Eswatini, Population Services International (PSI), in collaboration with the United States Centers for Disease Control and Prevention (CDC) and the Eswatini Ministry of Health, implemented a community-based HIV testing, mobile HIV-care, peer-delivered linkage case-management demonstration project (CommLink) [7] in 2015. CommLink provided a comprehensive package of linkage services for up to 90 days in accordance with CDC and WHO recommendations [11,12].

The information on the costs of providing a comprehensive package of recommended peer-delivered linkage services is limited, and the costs of retention on ART associated with these services have not been reported. In this paper, we present the estimated costs of the HIV diagnosis, ART initiation, and ART retention (HIV-care) cascade among CommLink clients diagnosed during 2016–2018, before Eswatini achieved 95-95-95. Studies of interventions delivered in high-HIV-burden countries that are approaching 95-95-95 are particularly important given the expected higher costs to test, diagnose, and treat the remaining potentially hardest-to-find population of PLHIV in need of ART. Moreover, although often more costly than facility-based services, community-based services are critical during the COVID-19 pandemic to sustain HIV testing and treatment when access to facilities is reduced or suspended because of lockdowns. Findings from this study, then, might help inform planning, budgeting, and implementing similar community-based linkage programs for high-burden countries approaching 95-95-95.

## 2. Materials and Methods

For full details of the CommLink description and methods of the retrospective cohort study, see [7,8,11]. Briefly, PSI implemented community-based mobile and index HIV testing services following the national guidelines from 2015 to 2018. Peer expert client counselors provided services for up to 90 days to consenting HIV-positive clients identified at homesteads, worksites, bars, and other community locations in three of the four regions of Eswatini.

We conducted a retrospective cohort study to assess the program costs and clinical outcomes of CommLink in 2 urban and 13 rural Tinkhundla (i.e., constituencies) of the Manzini region over the implementation period. Clients who tested HIV-positive in the Manzini region between 1 March 2016 and 31 March 2018, aged > 15 years, had not received HIV care in the last 90 days, consented for follow-up services, and were referred to healthcare facilities in Manzini or in regional border zones were eligible for the retrospective cohort study. 

### 2.1. Clinical Outcomes

In this paper, we focus on the clinical outcomes on the clients who tested HIV-positive, HIV-positive clients who were eligible for and participated in CommLink, and CommLink clients who initiated and remained on ART. ART initiation was defined as receiving ART at least once on or after the HIV diagnosis date. Retention was defined as having a clinic visit within 90 days after the last antiretroviral refill appointment [13]. Using the test records from referral facilities and regional and national ART databases during July 2019–April 2020, abstraction teams located and matched eligible clients to abstract the outcome data. We used generalized estimating equations (GEE) models with a log link (SAS 9.4) to estimate ART initiation and retention outcomes [8]. Separate GEE models adjusting for age, sex, geographic area, and within-Tinkhundla clustering were conducted to estimate the number of clients initiated on ART, and the number of clients initiated and retained on ART (combined outcome).

### 2.2. Cost Data

Program costs associated with CommLink services and study clinical outcomes were collected from a provider perspective using an ingredients-based approach by cost category (personnel, commodities and supplies, travel, and training), and by program activity (testing services (i.e., point-of-diagnosis medical assessment, peer-delivered counseling, and testing of partners and family members), linkage services (i.e., follow-up face-to-face HIV counseling, psychosocial-support, and escorting), retention services (i.e., treatment navigation services, telephone support, and appointment reminders), mentoring and supervision, and monitoring and evaluation (M&E)). Mentoring and supervision activities included meetings with team leaders or supervisors to review open cases, troubleshooting and managing difficult cases, and modifying case-management plans as needed. M&E included completing program forms, registers, logs, or daily diaries. Capital costs (training, vehicle, and mobile unit) were annuitized over the estimated useful life of each cost item, with a discount rate of 3%, consistent with accepted methods [14]. 

Cost data were collected retrospectively from financial reports and interviews with HIV test counselors, expert client counselors, M&E officers, nurses, managers, referral and linkage coordinators, supervisors, and the PSI finance team. Data were collected in Swazi lilangeni (SZL), adjusted by the proportion of Manzini clients eligible for the retrospective study, inflated to 2018 price levels using the Eswatini consumer price index, and converted to USD using the average market exchange rate in 2018 [15,16]. Cost data categorized by service type were used to calculate cost-to-outcome ratios for testing HIV-positive, CommLink eligibility and participation, ART initiation, and combined ART initiation and retention. All cost analyses were performed using Microsoft Excel for Office 365.

### 2.3. Scenario Analysis

Because most PLHIV in Eswatini were currently receiving HIV care and ART by 2017 [2], CommLink expert client counselors were not maximally employed during the study period, having on average 10 clients to manage at any given time. We therefore estimated costs assuming a scenario of improved productivity based on two related observations. First, CommLink expert client counselors and supervisory staff reported being able to manage at least 20 clients at any time without reducing services if the program restricted services to clients who received care in Manzini region. Providing case-management services to clients who resided in other regions was a substantial burden on CommLink staff, the majority of whom lived in Manzini. Second, a reduced service model of CommLink was approved by the Eswatini Ministry of Health and scaled up by PSI in all four regions of Eswatini in 2019 [17]. Under the national model, regional case-management services are restricted to patients who receive care in regional clinics. Additionally, the reduced national model requires only one follow-up meeting with clients (rather than two required by CommLink). Based on 2019 case reviews (conducted by authors DM and DW), the average number of clients actively managed by PSI expert client counselors often exceeded 30 cases under the national model. The scenario analysis, therefore, assumed that the full package of CommLink services could have been provided on average to at least 20 Manzini clients per expert client counselor at any given time. 

## 3. Results

During the study period, CommLink had 4 outreach teams and the following 32 staff (full time and part time): 4 HIV-test counselors (one per team), 4 nurses (one per team), 19 expert client counselors (4–5 per team), 2 part-time M&E data-entry clerks, 2 expert-client mentors, and a linkage and retention coordinator. During outreach testing events conducted in Manzini region, we estimated that 875 (80%) of 1089 clients who tested HIV-positive were eligible for CommLink. Of those eligible, 822 (94%) participated in CommLink, of whom 773 were eligible for and included in the retrospective study (8). By the end of the study follow-up period (median, 961 days; interquartile range, 827–1093 days), 97% of clients had initiated ART, and 76% of clients were initiated and retained on ART eighteen months after diagnosis (Table 1).

**Figure 1:**
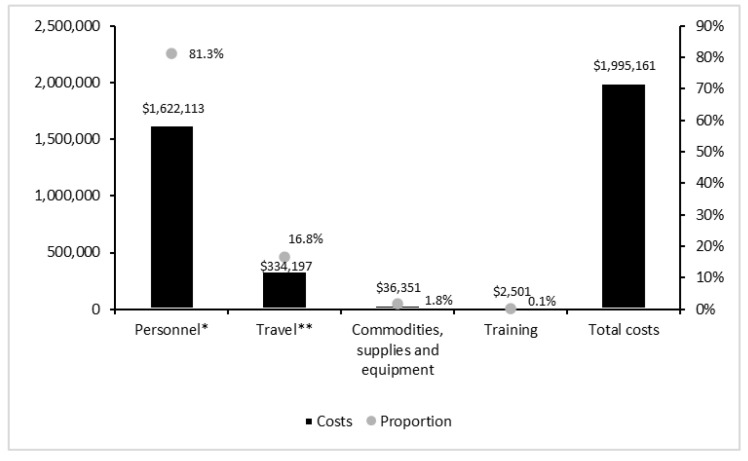
Total program costs by cost category (in 2018 USD). * Wages, salaries, bonuses, and allowances. ** Vehicles, mobile units, drivers, and transport payments made to staff.

**Figure 2:**
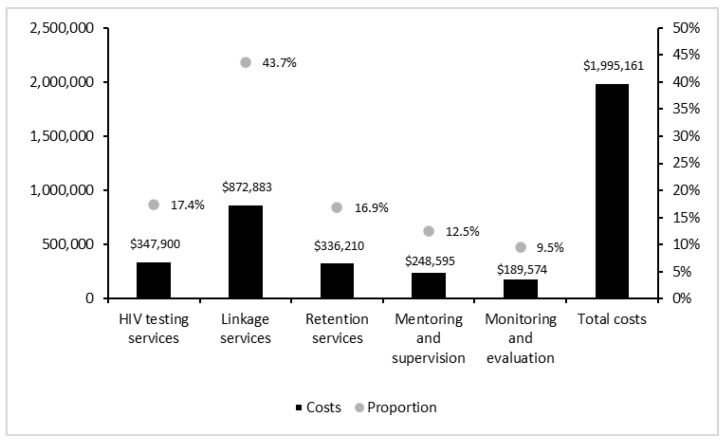
Total program costs by program activity (in 2018 USD).

Estimated total program costs were approximately $2 million (Figure 1 and Figure 2). Personnel costs were the dominant cost component (81%), followed by travel (17%), commodities and supplies (2%), and training (0.1%) (Figure 1). About 61% of total costs were associated with linkage (44%) and retention (17%) services, whereas costs associated with testing services accounted for 17% of total costs (Figure 2). 

The total costs for testing, linkage, and retention services inclusive of mentoring and supervision and M&E were $542,875 (27%), $1,011,461 (51%), and $440,826 (22%), respectively. Table 2 presents ratios of cost per study outcome. The costs per client tested positive, per client eligible for CommLink, and per CommLink participant were $499, $620, and $660, respectively. The estimated cost per client ever initiated on ART was $1354, ranging from $2114 to $1480 for clients initiated on ART within 7–90 days of diagnosis (Table 2). The estimated cost per client initiated and retained on ART 6 months after diagnosis ($2343) increased slightly for clients who were initiated and retained on ART 18 months after diagnosis ($2462).

Table 3 presents a scenario analysis assuming that CommLink expert client counselors would be more fully employed, managing approximately 20 clients at any given time. The total costs for CommLink would be reduced by $390,191 and result in lower costs to outcome ratios ($1090 for overall ART initiated, $1191 for ART initiated within 90 days, and $1873 for initiated and retained at 18 months).

## 4. Discussion

Our study provides the first estimated HIV-care-cascade costs of a community-based, peer-delivered linkage case-management intervention. The cost per client tested HIV-positive ($499, inflation adjusted 2018 USD) for CommLink is consistent with those reported from other community-based testing studies conducted in sub-Saharan Africa (ranging from $65 to $1411 for home-based testing, and from $584 to $1114 for mobile testing) [18,19]. Similar to our findings in Eswatini, community-based HIV testing studies in South Africa and Botswana that have similar HIV epidemics estimated that costs per person diagnosed were $812 and $685, respectively [20,21]. While costs to identify HIV-positive clients in community settings are important, assessing costs to successfully initiate and retain these clients on ART is particularly important as few (<50%) clients enroll early in care in the absence of linkage services [7,8,9,10].

Costs on linkage services to help clients initiate ART are scanty and outdated. Previous studies on the costs of community-based services in Sub-Saharan Africa reported costs per patient linked to care (inflation adjusted 2018 USD) of $49 to $2231 [9,20,22,23]. The cost estimates per CommLink client initiated on ART ($1354) are consistent with a prior study conducted in Eswatini [9], and two studies are in countries in the same region with similar HIV epidemics [20,22]. The prior Eswatini study estimated the total costs of a mobile-testing strategy to link HIV-positive clients to care within 6 months of diagnosis to be $1831 USD [9]. Studies in Lesotho and South Africa estimated the total costs of mobile-testing services to link HIV-positive persons to care were $1147 and $2231 USD, respectively [20,22]. These studies, however, were conducted from 2011 to 2014, before the test and start era when all HIV-positive patients were eligible for ART, and they did not estimate the costs per client initiated and retained on ART. Although the programs and costing approaches are not directly comparable across studies due to different time periods, study designs, and cost definitions, CommLink appears to be relatively more cost-effective at achieving early ART initiation (90 days). Finding the remaining HIV-positive persons and linking them to ART may also cost more in Eswatini, as an estimated 96% of PLHIV in Eswatini were receiving ART in 2019 [2]. Notably, CommLink costs to initiate clients on ART within 7 days of diagnosis in accordance with WHO recommendations [24] were much higher ($2114) compared with 90 days of diagnosis ($1480). 

The costs of linkage services for ART initiation and retention have not been reported. CommLink retention services provided for up to 90 days (e.g., follow-up treatment navigation and supportive services) cost $2462 to help clients initiate and remain on ART 18 months after diagnosis. With a marginal cost increase of approximately $1108 USD compared with ART initiation ($1354), a comprehensive package of recommended linkage services that might also improve ART retention may be worth the investment. As countries strive to achieve 95-95-95, a higher per-person cost may be well justified to reach, test, and initiate and retain on ART the remaining HIV-positive clients who are likely the hardest to reach [25]. Health personnel are an important contributor to the costs of community-based linkage and retention services. Findings from our scenario analysis suggest that a more efficient community-based linkage case-management model is possible and that programs need to consider appropriate staffing levels to improve the use of limited resources. Programs considering a linkage case-management model similar to CommLink should consider initial staffing levels that result in an average open case load of approximately 20 clients per counselor. 

Our study has several important limitations. First, since this report is limited to a program evaluation of CommLink, we recognize that self-selection bias may have occurred, and some outcomes were compared with prior reports [7,8,9,10] could be attributed to unmeasured confounding. However, our results were adjusted for several important confounders, including age, sex, and urban or rural residence [8]. Second, the combined ART initiation and retention estimated in our study may be attributed, in part, to other interventions. The provision and nature of retention services provided after CommLink services ended (e.g., standard facility-based adherence counseling, psychosocial support, and defaulter tracing) were not measured and are unknown. Third, the costs of facility-based ART services were not captured in our study. Thus, estimated CommLink costs substantially underestimate the total costs for initiating and retaining clients on ART. Fourth, as a demonstration project that included start-up costs as well as extensive M&E, and mentoring and supervision costs, CommLink would likely have higher costs than mature, routinely implemented programs. Fifth, we did not collect costs at the client level, which might have provided insights into potential cost-related barriers to care and the benefits of using mobile units in bringing services to clients. Cost estimates, therefore, may not reflect the actual resources required to provide services by patient type. Finally, the generalizability of our findings to other populations is unclear, and caution should be applied when interpreting or applying our findings to other settings.

Despite these limitations, our economic findings are novel and may help inform decisions and strategies related to implementing community-based interventions to improve HIV testing, and early ART initiation and retention. Future research to assess the costs and cost-effectiveness associated with testing, linkage, and retention services for all providers (facilities and communities) by patient type is needed to help identify efficient options to improve the impact of limited HIV investments. 

## 5. Conclusions

Community-based programs can contribute to improving HIV diagnosis and uptake of ART, and cost information is important to guide scale up and to sustain effective programs. Considering the economies of scope in designing and delivering community-based services for the HIV-care cascade (from community HIV diagnosis through retention on ART) may lower overall HIV program costs and make services more cost-effective. Economic evidence of new scalable interventions, such as CommLink, that addresses the HIV-care cascade may help inform linkage strategies and explore options to strengthen effectiveness and improve efficiency. 

## Figures and Tables

**Table 1:** CommLink enrollment, ART initiation, and ART retention outcomes, Manzini Region, Eswatini, March 2016–March 2018 ^a^.

Outcomes	Number of Clients (%)
Estimated number of clients who tested HIV-positive	1089
Estimated number of clients eligible for CommLink ^b^	875
CommLink participants	822
CommLink participants in retrospective study ^c^	773
ART initiation after diagnosis ^d^	
Within 7 days	478 (62%)
Within 30 days	619 (80%)
Within 90 days	683 (88%)
Ever	747 (97%)
ART initiated and retained after diagnosis ^d^	
At 6 months	620 (80%)
At 12 months	611 (79%)
At 18 months	590 (76%)

^a^ HIV-positive clients identified in 2 urban and 13 rural Tinkhundla of Manzini region; 243 clients participated in CommLink from 1 March 2016 to 30 September 2016 (CD4 eligibility criteria of ≤500/μL); and 530 clients participated in CommLink from 1 October 2016 to 31 March 2018 (during test and treat). ^b^ Clients who received HIV care in the past 90 days or wished to be referred to distant health care facilities in other regions of Eswatini or other countries were not eligible for CommLink case-management services. ^c^ Restricted to clients aged >15 years referred to healthcare facilities in Manzini or in regional border zones. ^d^ Estimated using GEE.

**Table 2:** Costs per outcome (in 2018 USD).

	USD
Cost per client who tested positive ^a^	498.51
Cost per client who was eligible for CommLink ^b^	620.43
Cost per CommLink participant ^c^	660.43
Cost per ART-initiated client	1354.54
Cost per ART-initiated client within 7 days	2113.87
Cost per ART-initiated client within 30 days	1633.57
Cost per ART-initiated client within 90 days	1480.19
Cost per ART-initiated and -retained client at 6 months	2342.60
Cost per ART-initiated and -retained client at 12 months	2378.19
Cost per ART-initiated and -retained client at 18 months	2462.34

^a^ Cost associated with testing services, divided by the number of estimated clients tested HIV-positive. ^b^ Cost associated with testing services, divided by the number of estimated clients eligible for CommLink. ^c^ Cost associated with testing services, divided by the number of estimated clients consented to participate in CommLink.

**Table 3:** Costs ^a^ per outcome for scenario analysis (in 2018 USD).

	USD
Cost per ART-initiated client	1090.00
Cost per ART-initiated client within 7 days	1701.04
Cost per ART-initiated client within 30 days	1314.54
Cost per ART-initiated client within 90 days	1191.11
Cost per ART-initiated and -retained client at 6 months	1781.83
Cost per ART-initiated and -retained client at 12 months	1808.89
Cost per ART-initiated and -retained client at 18 months	1872.90

^a^ Total cost for linkage and retention services inclusive of mentoring and supervision and M&E was $813,925 and $290,711, respectively.

## Data Availability

Data regarding this study can be provided upon request to the corresponding author. Data are governed by the Eswatini Health Research Review Board (EHRRB) and are not publicly available to protect patient confidentiality. Datasets may be made available with EHRRB approval.
